# Can internet use change sport participation behavior among residents? Evidence from the 2017 Chinese General Social Survey

**DOI:** 10.3389/fpubh.2022.837911

**Published:** 2022-10-19

**Authors:** Hua-mei Zhong, Han-bing Xu, En-kai Guo, Jing Li, Zhao-hong Wang

**Affiliations:** ^1^School of Physical Education and Sport Science, Fujian Normal University, Fuzhou, China; ^2^College of Physical Education and Sports, Beijing Normal University, Beijing, China

**Keywords:** internet use, sport participation, probit model, impact mechanism, healthy China

## Abstract

**Purpose:**

The popularization of the internet has promoted the implementation of China's national fitness strategy and created conditions for Chinese residents to participate in sports. The internet is an essential medium for disseminating sports knowledge, and the use of the internet can change sport participation behaviors. Therefore, the internet can be used to popularize sports knowledge and promote the participation of all people in sports and thus improve the health of the entire population. This study attempts to empirically analyse how the use of the internet changes sport participation behavior.

**Method:**

Utilizing data from the 2017 China General Social Survey, a probit model, ivprobit model, and bias-corrected non-parametric percentile bootstrap test were used to analyse the impact of internet use on sport participation behavior.

**Results:**

The empirical results show that internet use significantly increased the probability of participation in sports by Chinese residents. Heterogeneity test results showed that internet use was more effective in promoting sport participation in middle-aged groups, groups of older persons, unmarried groups, and groups with a high school education or above. The mediating effect test results showed that internet use influenced residents' participation in sports by promoting social interaction, leisure and entertainment, and learning and recharging.

**Conclusions:**

The internet has changed participation in sports; specifically, the use of the internet promotes sport participation. Additionally, internet use has a more obvious impact on improving the sport participation behavior of middle-aged, older, unmarried, and middle- and higher-educated individuals. Internet social interaction, internet entertainment and internet learning are effective channels to encourage Chinese residents to participate in sports and improve their health.

## Introduction

Building a healthy China is a key task in China's socioeconomic development in the modern period. Implementing a national fitness strategy to increase the sport participation rate is an effective measure for promoting national health and health in China. However, China's population currently has a low participation rate in sports, creating challenges for the role of sports in promoting a healthy China. In 2014, only 33.9% of China's total population (including teenagers) participated in sports regularly (1). In 2018, the participation rate of Chinese residents in sports was only 30.9%, and the average time that residents participated in sports per day was only 31 min (2). In 2020, 37.3% of China's population over the age of 7 participated in sports regularly (3). In other countries, the sport participation rate of the population is significantly higher than that of China. According to statistics, in 2016–2017, 60.6% of the total population over the age of 16 in the UK participated in sports regularly, while in 2016, 61.8% of the population over the age of 15 in Australia played sports regularly (4). In 2017, 72% of the US population aged 6 or older was active in sports (5). These findings demonstrate the need to expand the number of Chinese people who participate in sports to improve the country's health.

As an important media tool to change lifestyles, the internet is often used in sports, and individuals' sports lifestyles are digitized, socialized, and personalized. The internet improves residents' awareness of sports, diversifies the participants involved in sports, and changes the conditions and forms of participation in sports. By December 2020, the number of netizens in China reached 989 million, and the internet penetration rate reached 70.4% (6). Against the background of promoting the construction of a healthy China and national fitness as a national strategy, the approach of “Internet + Sport” has made the national fitness approach smarter and changed the way residents participate in sports. At the same time, many Chinese policies regard the internet as an important measure to promote and improve the participation of all people in sports. The State Council's “Opinions on the Implementation of Healthy China Action” [(7) No. 13] proposed accelerating the deep integration of the internet, big data, artificial intelligence, and the physical economy of sports as well as innovating production modes, service modes and business models. The General Office of the State Council's “Opinions on Strengthening the Construction of National Fitness Facilities and Development of Mass Sports” [(8) No. 36] proposed actively promoting “Internet + Fitness” to improve the intelligence, information and digital level of national fitness public services. With the increasing number of internet users in China and the deep integration of Internet + Sport, the internet has become an effective measure to promote the implementation of the national fitness strategy and the construction of a healthy China. Does internet use have an impact on residents' sport participation behavior? Based on data from the Chinese General Social Survey in 2017, this study empirically examined the impact of internet use on residents' sport participation behavior and explored group heterogeneity and the influence mechanism of internet use on residents' sport participation to provide a research basis for improving sport participation and promoting national health.

## Literature review

### The internet and sport participation

With the continuous improvement of internet infrastructure and the popularization of the internet, the internet has become an important tool to change residents' lifestyles and impact their sport participation behavior. Relevant research shows that the use of the internet has a promotional effect on sport participation behavior, and the use of the internet and mobile phones for physical activity can significantly increase the health of adults and help maintain their physical activity levels (9). There is a positive correlation between internet use and a moderate amount of physical activity (10). Family exercise facilitated by the internet to promote social support and community centers can motivate older individuals to participate in physical activity (11). People who use the internet are more likely to participate in vigorous exercise, muscle-strengthening sport participation, and moderate physical activity (12). Using a pacemaker as a measuring tool, internet-based motivational interventions (IMIs) are effective in increasing physical activity among college students (13). Relevant studies in China also show that sports apps can significantly promote sport participation behavior and habit formation (14). The high frequency of internet use has a significant positive impact on sport participation in the rural floating population (15). The use of smartphone sports software can significantly improve the goal and behavioral attitudes as well as behavioral intention and other attitude-related variables of college students' sport participation (16).

Internet use has a positive effect on sport participation behavior. However, some studies show that internet use is not conducive to individual participation in sports or that there is no correlation between internet use and sport participation. Studies have shown that the use of computers at discretionary times among young people aged 18–30 was negatively correlated with physical activity (17), and there was no correlation between leisure internet time and leisure physical activity among African American adolescent girls (18). Printed sports interventions were more effective than internet sports interventions in increasing the intention and behavior of female adolescents to participate in sports activities (19). Furthermore, leisure time computer or internet use was not associated with leisure time physical activity levels (20). The high internet access rate of Taiwanese adolescents has been related to increased BMI, and activities related to the internet and mobile phones have been associated with an increase in BMI (21). Less participation in sports among adolescents has been significantly correlated with frequent internet use (22). Children who used mobile internet tablets had a 17% decrease in physical activity intensity and a 54% increase in sedentary behavior (23).

It can be seen from the above research results that the use of the internet or mobile internet not only promotes the participation of heterogeneous groups in sports activities but also has no correlation with the inhibition of sports activities. At the same time, research shows that excessive time spent online, internet cafes, and the use of the internet to vent emotions are related to adolescents' negative living habits even though the use of the internet to acquire knowledge and information has a positive predictive effect on healthy lifestyles for adolescents (24). The integration of the internet and sports has changed sports lifestyles and promoted participation in sports by popularizing health knowledge. Based on this, the first hypothesis is proposed:

**Hypothesis 1**: Internet use significantly improves the probability of participation in sports.

### Group differences in the use of the internet impacting sport participation behavior

Promoting the participation of all people in sports and improving health has become an important issue of concern to all countries in the world. China has launched the “National Fitness Plan” and the “Healthy China 2030” strategy to promote the active participation of all people in sports and improve health. From the perspective of participation demand, groups of different genders, ages, marriage status, and education have significant differences in sport participation demand. Studies have shown that the probability of males participating in sports is significantly higher than that of females (25–27). However, some studies have found the opposite result, suggesting that the probability of females participating in sports is significantly higher than that of males (28). The demand for sport participation by males is higher than that for females, which might be caused by the different sport participation preferences generated by gender. In addition, some studies have shown that age decreases the probability and duration of sport participation (27, 29–31), while other studies show a non-linear relationship between age and sport participation. Further research results show a U-shaped relationship between age and the sport participation rate (32–34). In comparison, Wicker et al. showed an inverted U-shaped relationship between age and sports club participation rates (35). While some studies have shown that married people are less likely to participate in sports than unmarried people (27, 29), others show that the probability of married people participating in sports is higher than that of unmarried people (28). The higher people's education level is, the higher their demand for sport participation such that higher education levels show a higher possibility of sport participation (25), more time spent participating in sports (33), and a higher frequency of sport participation (36). There are substantial differences in the level of social and economic development between urban and rural areas in China. At the same time, the internet penetration rate in rural China is lower than that in urban China. Are there gender, age, marriage, education and urban and rural differences in the impact of internet use on residents' sport participation behaviors? Based on this question, the second research hypothesis is proposed.

**Hypothesis 2**: There are differences in gender, age, marriage, education, urban and rural areas in the impact of internet use on residents' sport participation behaviors.

### The mechanisms of the internet that impact sport participation

As an information communication platform mediated by computer and mobile communication tools, the internet is an emerging information and communication medium. At the same time, the internet has become an important vehicle for Chinese residents to engage in social interaction, leisure, and recreation in their spare time and to invigorate their studies. According to the survey report of the China Internet Network Information Center (CNNIC), as of December 2020, 978.44 million residents in China used mobile instant messaging and 516.37 million residents used mobile online games or other recreational activities. The number of residents using mobile online education reached 340.73 million (6). First, internet information dissemination promotes individual participation in sports by promoting social communication. He found that the internet broadens the channels through which individuals gather information and strengthens their social networks (37). At the same time, interpersonal communication using the internet is characterized by “super interpersonal communication”, which is conducive to flexible communication between people and the formation of daily interpersonal communication (38). Therefore, due to the popularity of the internet, especially the mobile internet, and the efficiency of information transmission, individuals use the internet as an information transmission medium to promote social communication between individuals, and the formation of social networks promotes individual participation in sports through the mutual dissemination of sports information. Second, internet information dissemination promotes individual participation in sports by promoting widespread participation in leisure and entertainment activities. The integration of the “game” attribute of sports and the internet is inspired by sports communication. Internet + Sport optimizes mass sport participation and organizational modes to promote sport participation offline (39). Watching sports programmes is one of the important avenues for leisure and entertainment. In their spare time, individuals can spread sports culture to improve the sports awareness of the population by watching live sporting competitions, thus promoting individual participation in sports. Third, internet information dissemination promotes individual participation in sports by promoting participation in learning. The acquisition of sports knowledge is essential to encourage individuals to improve their health cognition. Numerous studies have found that participation in sports promotes health (40) and is an important means to improve individuals' health. With the deep integration of sports and the internet, residents can obtain sports training similar to “teaching by words and deeds” without leaving home. In their spare time, residents can gain health knowledge and sport skills through fitness apps, thereby promoting individual participation in sports. In summary, residents use the internet in their spare time for social communication, recreation, learning, and recharging, all of which are important channels for participation in sports. Therefore, the third research hypothesis is proposed.

**Hypothesis 3**: Social interaction, leisure and entertainment, and learning and recharging are the intermediary mechanisms of internet use that impact participation in sports; that is, internet use promotes residents' participation in sports by affecting residents' social interaction, leisure and entertainment, and learning and recharging.

## Methods

### Measure of variables

#### Sport participation

“Participation in sports within a week” was used to measure individual sport participation behavior, a dichotomous variable. The source item was the 2017 Chinese General Social Survey (CGSS2017) questionnaire A15a: “In the past 12 months, how many times per week did you engage in sport participation activities that normally lasted 30 min and made you sweat?” The answer “0 times” was assigned a value of 0 (does not participate in sports), while a value of 1 was assigned to the answer “once or more” (i.e., participates in sports). Of the 10,688 participants, 4,616 participated in sports every week, accounting for 43.19%.

#### Internet use

The questionnaire to measure internet use was A285, with the item “Your use of the following media in the past year—internet (including mobile internet)”. The answers “never” and “rarely” were assigned a value of 0 (no internet use), while “sometimes,” “often,” and “very often” were assigned values of 1, indicating the use of the internet. Internet use occupies a large amount of residents' sport participation time. Therefore, the frequency of internet use is taken as the independent variable in this study to verify whether there is a non-linear relationship between the frequency of internet use and sport participation rates. The answers “never,” “rarely,” “sometimes,” “often,” and “very often” were assigned values from 1 to 5, respectively. A robustness test was conducted using alternative variables, and “surfing the internet during idle time” and “frequency of surfing the internet during idle time” were taken as alternative variables. The questionnaire to measure internet use during free time was A3012; the answers “never” and “a few times a year or less” were assigned a value of 0, and the rest of the answers were assigned a value of 1. Answers to the question “How often do you use the internet in your free time?” were assigned values ranging from 1 to 5.

To verify whether social interaction, leisure and entertainment, and learning and recharging are mediating mechanisms of internet use that affect residents' participation in sports, social interaction, leisure and entertainment, and learning and recharging were selected as the mediating variables for verification. Answers to the question, “In the past year, have you done any of the following things in your free time?” included socializing/visiting, relaxing and learning. The answers “never,” “rarely,” “sometimes,” “often,” and “very frequently” were assigned values from 1 to 5, respectively, to measure the social interaction, leisure and entertainment, and learning and recharging status of residents during leisure time. To reduce the number of missing variables, we estimated the result deviation. According to the research results, gender, age, marriage, urban and rural areas, education, political affiliation, BMI, health, life happiness, social class, individual income and family economic conditions were used as control variables, and the level of regional economic development and regional characteristics were controlled for.

### Data collection

This study is based on the 2017 Chinese General Social Survey (CGSS) and was conducted to empirically verify whether internet use can promote residents' participation in sports and verify the heterogeneity and related mechanisms of the impact. This study provides a research basis for the Internet + Sports development strategy and the implementation of the “national fitness strategy”. In 2017, for the first time, the China General Social Survey Project investigated the amount of sport participation by Chinese residents in a week, providing a reliable sample for this study. The total sample size of CGSS2017 was 12,582, and 10,688 valid samples were generated after removing the missing values and selecting relevant variables. The per capita GDP data came from the China Statistical Yearbook 2018 and were matched with survey data by province codes.

### Analytical method

As the dependent variable, sport participation was a binary variable. A probit model was adopted to analyse the impact of internet use on residents' sport participation behavior, and a baseline model was established to test the impact of internet use on residents' sport participation and the non-linear relationship between internet use frequency and residents' sport participation. A robust standard error was used for estimation, and marginal effect coefficients were reported: *SP*_*i*_ is sport participation, internet_*i*_ is internet use, and *Control*_*i*_ is the control variable.


$$\begin{array}{l} Pr\;\left(SP_{i}=1\right)=\Phi \left(\alpha_{1}Internet_{i}+\beta^{\prime }Control_{i}+\varepsilon_{i}\right) \end{array}$$

To further test whether social interaction, leisure and entertainment, and learning and recharging are the mediating pathways of internet use that affect residents' participation in sports, a non-parametric percentile bootstrap test with bias correction was used to test the mediating effect; 1,000 samples were repeated, and the mediating effect value of the 95% confidence interval was calculated.

## Results

### Descriptive statistics

**Figure 1:**
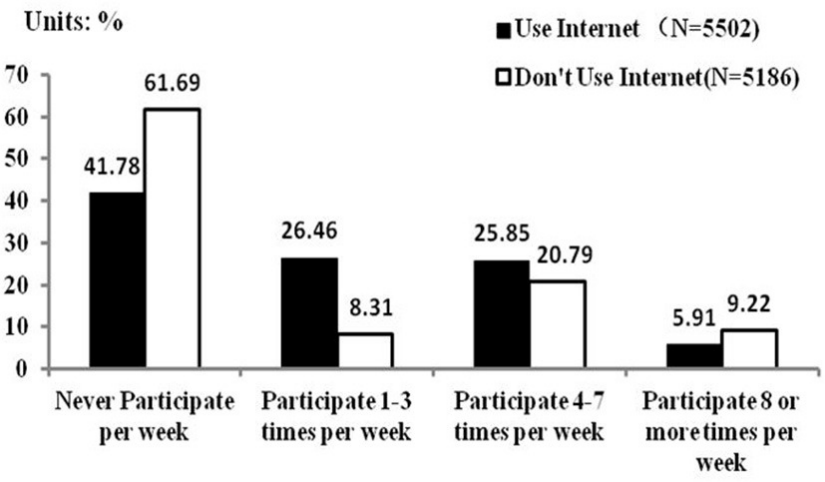
Sport participation rate of residents using/not using the internet.

Of the 10,688 samples, 5,502 used the internet, accounting for 51.49%. This is essentially consistent with the 55.8% internet penetration rate in 2017 in the 41st Statistical Report on Internet Development in China in 2018 (41). Figure 1 shows that 41.78% of residents who use the internet do not exercise weekly, while 61.69% of residents who do not use the internet do not exercise weekly. The proportion of residents who use the internet to exercise 1–3 times a week and 4–7 times a week is higher than those who do not use the internet. However, the proportion of residents who do not use the internet to exercise 8 times or more per week is higher than that of residents who use the internet. The precise measurement methods and descriptive statistical results of the relevant variables are shown in Table 1.

**Table 1:** Variable names, variable definitions, and descriptive statistics [mean (SD)/*n* (%)].

**Variable**	**Use internet**	**Not use internet**	***p-*value**
**Sport participation**			
Yes	3,002 (54.56)	1,614 (31.12)	*0.000*
No	2,500 (45.44)	3,572 (68.88)	
**Gender**			
Male	2,689 (48.87)	2,412 (46.51)	*0.014*
Female	2,813 (51.13)	2,774 (53.49)	
**Age**	41.46 (0.19)	61.67 (0.17)	*0.000*
**Marriage**			
Married	4,149 (75.41)	3,985 (76.84)	*0.083*
Unmarried	1,353 (24.59)	1,201 (23.16)	
**Village type**			
Urban	4,321 (78.54)	2,448 (47.20)	*0.000*
Rural	1,181 (21.46)	2,738 (52.80)	
**Education**			
Primary school and below	606 (11.03)	3,131 (60.37)	*0.000*
Junior middle school	1,622 (29.48)	1,440 (27.77)	
Senior high school (technical school)	1,373 (24.95)	492 (9.49)	
College	1,761 (32.01)	122 (2.35)	
College or above	139 (2.53)	1 (0.02)	
**Political identity**			
Member of Communist Party of China	771 (14.01)	424 (8.18)	*0.000*
Others	4,731 (85.99)	4,762 (91.82)	
**BMI index**	22.76 (0.05)	22.71 (0.05)	*0.263*
**Health level**			
Very unhealthy	86 (1.56)	419 (8.08)	*0.000*
Relatively unhealthy	378 (6.87)	1,368 (26.38)	
Generally	1,289 (23.43)	1,474 (28.42)	
Relatively healthy	2,284 (41.51)	1,465 (28.25)	
Very healthy	1,465 (26.63)	460 (8.87)	
**Life happiness** (**0–10)**	2.27 (0.05)	2.08 (0.05)	*0.000*
**Social class**			
Lower	930 (16.90)	1,624 (31.32)	*0.000*
Middle lower	2,039 (37.06)	1,773 (34.19)	
Middle	2,163 (39.31)	1,597 (30.79)	
Middle upper	360 (6.54)	178 (3.43)	
Upper	10 (0.18)	14 (0.27)	
**Personal income**	9.03 (0.05)	7.62 (0.05)	*0.000*
**Family economic**			
Well below average	243 (4.42)	663 (12.78)	*0.000*
Below average	1,732 (31.48)	2,151 (41.48)	
Average	3,016 (54.82)	2,111 (40.71)	
Above average	492 (8.94)	246 (4.74)	
Well above average	19 (0.35)	15 (0.29)	
**Regional economic development level**	11.13 (001)	10.97 (0.01)	*0.000*
**Region dummy variable**			
Western	977 (18.12)	1,166 (22.48)	*0.000*
Middle	1,483 (26.95)	1,988 (38.33)	
Eastern	3,022 (54.93)	2,032 (39.18)	
**Social interaction**			
Never	470 (8.54)	740 (14.27)	*0.000*
Rarely	1,777 (32.30)	1,804 (34.79)	
Sometimes	1,905 (34.62)	1,367 (26.36)	
Often	1,162 (21.12)	973 (18.76)	
Very often	188 (3.42)	302 (5.82)	
**Leisure and entertainment**			
Never	62 (1.13)	164 (3.16)	*0.000*
Rarely	641 (11.65)	724 (13.96)	
Sometimes	1,938 (35.22)	1,589 (30.64)	
Often	2,491 (45.27)	2,183 (42.09)	
Very often	370 (6.72)	526 (10.14)	
**Learning and recharging**			
Never	1,373 (24.95)	3,640 (70.19)	*0.000*
Rarely	1,713 (31.13)	982 (18.940	
Sometimes	1,378 (25.05)	314 (6.05)	
Often	862 (15.67)	184 (3.55)	
Very often	176 (3.20)	66 (1.27)	

The *p*-values in the table are the results of the *t*-test or chi-square test. The italic values indicate *p*-value less than 0.1 which are significant difference at the 90% confidence interval.

### Baseline regression

Following the empirical model established above, Stata 15.0 software was used for analysis, and the marginal effect of the independent variable on the dependent variable was calculated according to the regression coefficient. A variance inflation factor (VIF) was used to test the multicollinearity of the independent variables. The VIF values of the other independent variables were all < 3. Table 2 reports the impact of internet use on residents' participation in sports.

**Table 2:** Baseline regression: The impact of internet use on sport participation (SP).

**Variable**	**SP (Probit)**			
	**(1)**	**(2)**	**(3)**	**(4)**
Internet use	0.228*** (0.009)	0.071*** (0.013)		
Internet use frequency			0.071*** (0.002)	0.024*** (0.004)
Gender		0.003 (0.009)		0.003 (0.009)
Age		0.001*** (0.000)		0.002*** (0.000)
Marriage		−0.030*** (0.011)		−0.029*** (0.011)
Village type		0.126*** (0.011)		0.124*** (0.011)
Education		0.063*** (0.005)		0.061*** (0.005)
Political identity		0.064*** (0.015)		0.064*** (0.015)
BMI index		0.002* (0.001)		0.002* (0.001)
Health level		0.040*** (0.005)		0.039*** (0.005)
Life happiness		0.003** (0.001)		0.003** (0.001)
Social class		0.017*** (0.006)		0.017*** (0.006)
Personal income		−0.001 (0.001)		−0.001 (0.001)
Family economic		0.034*** (0.007)		0.033*** (0.007)
Regional economic development level		0.068*** (0.015)		0.065*** (0.015)
Region dummy variable		−0.024*** (0.008)		−0.024*** (0.008)
Prob > chi^2^	0.000	0.000	0.000	0.000
Pseudo R^2^	0.041	0.106	0.047	0.107
Observations	10,688	10,688	10,688	10,688

Marginal effect is reported in the table, i.e., dy/dx, and delta method standard error in parentheses.

*** *p* < 0.01,

** *p* < 0.05,

* *p* < 0.1.

In Model (1), the marginal effect of internet use on sport participation was 0.228 without adding any control variables and was significant at the 1% statistical level. Considering that other factors also impact participation in sports, after adding relevant control variables into Model (2), the marginal effect of internet use on sport participation was calculated as 0.071, which was significant at the statistical level of 1%. The results showed that the probability of residents participating in sports increased by 7.1% due to internet use. Model (3) and Model (4) report the non-linear relationship between the frequency of internet use and the sport participation rate. In Model (3), the marginal effect of the frequency of internet use on sport participation was 0.071. After the addition of control variables, the marginal effect of Model (4) was reduced to 0.024 and was significant at the statistical level of 1%. The results show that using the internet and increasing the frequency of internet use can improve residents' sport participation rate. Hypothesis 1 is verified: internet use significantly improves the probability of residents' participation in sports.

Among the control variables (except for gender, BMI and individual income), residents with advanced age, no marriage, urban residence, high education level, party membership, high health level, high sense of life happiness, high social class, and good family economic conditions had a higher probability of participating in sports.

### Endogenous processing: Instrumental variable approach

There may be endogeneity problems between internet use and sport participation, such as reverse causality between the two; people who participate in sports have more leisure time and are more likely to use the internet. Therefore, the ivprobit model was used to re-estimate the impact of internet use on residents' sport participation by using the ivprobit model as the instrumental variable for an individual who owns a mobile phone attributed to individual use alone and the number of internet access devices owned by an individual. The use of these two instrumental variables is based on the following considerations: with the popularity of mobile internet, individuals with mobile phones are more likely to use mobile internet to obtain information, and individuals with more internet access devices are more likely to use the internet. Table 3 estimates the estimation results of the supply variable method. The Wald test results show that Model (1) rejects the exogenous hypothesis at the 1% level, and Model (2) rejects the exogenous hypothesis at the 5% level, indicating that internet use endogenously affects residents' sport participation. At the same time, the F values of the first-stage regression are all higher than the empirical value of 10, and the regression coefficients of instrumental variables are statistically significant at the 1% level, indicating that there is not a weak instrumental variable problem. The estimation results of the instrumental variable method show that internet use significantly improves the probability of residents' sport participation, and the estimation results are consistent with the above results.

**Table 3:** Instrumental variable method: The impact of internet use on sport participation (SP).

**Variable**	**SP (IVProbit**)	
	**(1)**	**(2)**
Internet use	2.089*** (0.433)	0.874*** (0.253)
Control variable	Control	Control
Constant	−3.175*** (0.519)	−3.701*** (0.454)
Observations	10,688	10,688
Wald test	23.51	7.40
Prob > Chi^2^	0.000	0.007
**One stage regression results**	**Internet use**	
	**(1)**	**(2)**
Do you have a cell phone for your own use only?	0.121*** (0.012)	
Number of internet-connected devices		0.063*** (0.004)
Control variable	Control	Control
*F*-value in one stage	768.54	786.75

Regression coefficients are reported in the table, standard error is in parentheses.

*** *p* < 0.01.

### Robustness test: Replace independent variables

Obtaining sports information online is the primary purpose of internet use, and internet use is accompanied by sport participation. According to the above design, “leisure time online” and “leisure time online frequency” are used as alternative variables for internet use in conducting the robustness test. Table 4 reports the impact of online leisure time on residents' sport participation. The results show that the marginal effect of Model (1) of leisure time online and residents' sport participation is positive and significant at the 1% level. In Model (2), the marginal effect of leisure time online frequency on sport participation is 0.017 and significant at the 1% level. The results show that leisure time online significantly increased the probability of residents' sport participation. The robustness test results for the replacement of independent variables are consistent with the above results, indicating that the research results are reliable.

**Table 4:** Robustness test: The impact of leisure time online on sport participation (SP).

**Variable**	**SP (Probit)**	
	**(1)**	**(2)**
Leisure time online	0.062*** (0.013)	
Leisure time online frequency		0.017*** (0.003)
Control variable	Control	Control
Prob > chi^2^	0.000	0.000
Pseudo R^2^	0.106	0.106
Observations	10,688	10,688

Marginal effects are reported in the table, i.e., dy/dx, and delta-method standard error in parentheses.

*** *p* < 0.01.

### Heterogeneity analysis

There may be differences in the impact of internet use on sport participation among different groups. Therefore, interactive items of internet use with gender, age, marriage, education and village type are added to investigate the heterogeneity of internet use on residents' sport participation. According to the results of Table 5, the marginal effect estimates of internet use × gender vs. internet use × village type are 0.011 and −0.002, respectively, but there is no statistically significant level. The estimates show that the probability of females using the internet for sport participation is lower than that of males. In contrast, there is no significant difference in the use of the internet in relation to the probability of sport participation for urban dwellers compared to rural residents. The marginal effects of internet use × age, internet use × marriage, and internet use × education are 0.059, −0.095, and 0.050, respectively, and are significant at the 1% level. The results show that the probability of sport participation in middle-aged and older persons using the internet is significantly higher than in the younger group, the probability of sport participation in the married group using the internet is significantly lower than that in the unmarried group, and the probability of sport participation in the high school and above education group is significantly higher than that in the junior high school and below education group. The above results indicate that internet use plays a more significant role in promoting the participation of middle-aged groups and groups of older persons, unmarried groups, and groups with a high school education or above. That is, there are differences in age, marriage and education level in the influence of internet use on residents' participation in sports, while there are no differences in gender or urban and rural areas. Therefore, Hypothesis 2 is partially verified.

**Table 5:** Internet use impacts the heterogeneity of residents' sport participation (SP).

**Variable**	**SP (Probit)**				
	**(1)**	**(2)**	**(3)**	**(4)**	**(5)**
Internet use	0.066*** (0.015)	0.026 (0.018)	0.155*** (0.024)	0.058*** (0.013)	0.073*** (0.018)
Internet use × gender (Reference group: female)	0.011 (0.018)				
Internet use × age (Reference group: youth)		0.059*** (0.016)			
Internet use × marriage (Reference group: unmarried)			−0.095*** (0.023)		
Internet use × education (Reference group: Junior high school and below)				0.050*** (0.018)	
Internet use × village type (Reference group: rural)					−0.002 (0.020)
Control variable	Control	Control	Control	Control	Control
Prob > chi2	0.000	0.000	0.000	0.000	0.000
Pseudo R^2^	0.106	0.107	0.108	0.107	0.106
Observations	10,688	10,688	10,688	10,688	10,688

Marginal effects are reported in the table, i.e., dy/dx, and delta-method standard error in parentheses.

*** *p* < 0.01.

### Mechanism analysis

For internet use to clearly affect the sport participation mechanism such that internet use for recharging, social intercourse, entertainment, and learning impact residents' participation in sports, a deviation correction of non-parametric percentile bootstrap test methods was used to determine participation in the mediation mechanism. The inspection was repeated and sampled 1,000 times, and the 95% confidence interval was calculated for the intermediary effects value. The results (see Table 6) show that the indirect effects of internet use on residents' sport participation through social interaction, leisure and entertainment, learning and recharging are 0.005, 0.005, and 0.025, respectively. In contrast, the indirect effects account for 6.76, 6.76, and 32.43% of the total effects (0.074), respectively. In addition, 95% confidence intervals do not include 0, indicating that the mediation effect is established and partially mediated. The above results indicate that social interaction, leisure and entertainment, and learning recharges are important intermediary channels through which internet use influences participation in sports. In other words, internet use promotes residents' social interaction, leisure and entertainment, learning and recharging and influences residents' sport participation behavior. Hypothesis 3 is verified. It can be seen from the intermediary degree that internet use mainly influences residents' sport participation by promoting learning and recharging. The robustness test of “leisure time online” also obtained the same result.

**Table 6:** Test of the mediating mechanism of internet use impacting sport participation (SP).

**Mediating variable**		**Coefficient**	**Bootstrap standard error**	***P*>|z|**	**95% confidence interval**		**Degree of mediation**
					**Lower limit**	**Upper limit**	
Social interaction	Direct effect	0.005	0.001	0.000	0.002	0.007	6.76%
	Indirect effect	0.069	0.013	0.000	0.044	0.095	
Leisure and entertainment	Direct effect	0.005	0.001	0.000	0.003	0.007	6.76%
	Indirect effect	0.069	0.013	0.000	0.044	0.094	
Learning and recharging	Direct effect	0.024	0.003	0.000	0.019	0.030	32.43%
	Indirect effect	0.050	0.013	0.000	0.024	0.075	

The number of bootstrap resamples is 1,000.

## Discussion

The aforementioned research findings suggest that internet use can alter residents' sport participation behavior. This is evidenced by the fact that internet use significantly increases residents' likelihood of participating in sports and that this likelihood increases with residents' internet use frequency. The research results of Wang et al. (15) and Wang et al. (42) on the floating population and the elderly population are consistent but are inconsistent with the research results of Li et al. (43) on rural residents. The possible reason is that the sports information transmission provided by the internet improves health awareness and changes sport participation behavior. Although there are fewer opportunities to interact in person, using the internet has a favorable impact on people's participation in sports. This implies that internet use has altered people's attitudes toward participating in sports, which is beneficial for improving the level of national health. It is possible to employ “Internet + Sports” as a policy tool to enhance the health of Chinese citizens.

Internet use has a more obvious effect on promoting the participation of middle-aged and older people, unmarried people, and people with a high school education or above. From the perspective of age, people who use the internet are more likely to participate in sports if they are middle-aged or older. Health is a form of capital that depreciates with age, but engaging in sports can preserve or improve the stock of health. Therefore, one of the reasons why internet use encourages older individuals to participate in sports is because of their greater health needs. From the perspective of marital status, unmarried people who use the internet are more likely to participate in sports. Unmarried people are less involved in family work and family care, and their leisure time is mainly used on the internet and sports. However, there is a substitution effect for the amount of time spent participating in sports due to the increase in family work and caregiving in the married group. From the perspective of education level, higher education correlates with increased health demands and more frequent internet use. The internet provides rich scientific sports knowledge for people with higher education levels and encourages their sport participation behavior.

There is bidirectional causality between social interaction, leisure and entertainment, learning and recharging and internet use. On the one hand, people's social interaction, leisure and entertainment, and learning and recharging needs promote their internet use; on the other hand, internet use strengthens people's social interaction, leisure and entertainment, and learning and recharging needs. These research results show that internet use affects residents' participation in sports by promoting social interaction, leisure and entertainment, and learning and recharging. The impact of internet learning and recharging on residents' participation in sports is more obvious. The theory of communication games holds that communication allows people to immerse themselves in an action game, enjoy freedom in the game and generate pleasure in their own existence (44). The integration of the “game” property of sports and the internet gives internet sports communication the characteristics of sociability, entertainment and education. Individual sport participation motivation is generated through internet social interaction, internet entertainment and internet learning and then internalized into sport participation behavior. From the perspective of internet social interaction, sports, as a natural game, integrates the internet with social functions (e.g., instant messaging and social media) to immerse residents in social interaction in a virtual environment and create a social community with group aggregation and influence. When residents use internet sports products and services, they can realize social communication through internet social media in virtual networks and obtain personality shaping and value identification from sports as communication carriers, forming sport participation motivation and promoting the participation in sports offline. From the perspective of internet entertainment, the importance of the internet as a leisure entertainment platform along with the use of the mobile internet for leisure entertainment continues to grow. Sports competitions and performances provide important content and entertainment; through sports entertainment, national sports cognition and sports interest grow and promote national sport participation. From the internet learning perspective, sports information asymmetry is an obstacle that affects sport participation. Sports science, products and services are widely disseminated on the internet and among ordinary residents as consumers obtain sports information from the internet. Sport skills and knowledge form consumers' motivation for promoting offline sport participation. Internet leisure activities, entertainment, and learning have an impact on residents' participation in sports. In promoting the Internet + Sport depth fusion process, the need to optimize the internet social function, the entertainment function and the education function for national fitness is deepened to create a positive internet environment.

## Conclusion and recommendations

The internet has become an effective means to promote the implementation of the national fitness strategy. The deep integration of Internet + Sport has created conditions for residents to participate in sports and improve their physical health. Based on the data of the 2017 Chinese General Social Survey, this study adopts a probit model to empirically study the impact of internet use on residents' sport participation behavior and its mechanism at the micro level, indirectly verifying the effectiveness of the internet in promoting residents' health. The following conclusions are drawn. First, internet use significantly improves the probability of residents' sport participation. The instrumental variable method was used to solve endogeneity and robustness tests, and the results showed that this conclusion was valid. Second, heterogeneity test results showed that internet use had a more significant effect on promoting the participation of middle-aged groups and groups of older persons, unmarried groups, and groups with a high school education or above. Third, the mediating effect test results showed that social interaction, leisure and entertainment, and learning and recharging are important channels through which internet use affects residents' participation in sports.

The above conclusions reflect the practical significance of internet popularization in promoting the health of the population and promoting the construction of a healthy China. Combined with the research results, this study presents the following implications for promoting the deep integration of Internet + Sport and promoting the participation of the population in sports.

China urgently needs to promote the integrated development of the Internet + Sport and accelerate the digital transformation of sports products. The internet is a media tool that plays a key role in promoting sport participation; thus, it is necessary to actively promote the deep integration of the Internet + Sport and accelerate the digital transformation of sports products. This includes promoting the digital upgrading of sports products and services through the establishment of the Internet + Sport smart platform to provide personalized sports products and services that meet the sport participation demands of all people and provide scientific training guidance. At the same time, healthy internet use should be actively advocated. Excessive use of the internet (which affects residents' participation in sports) should be avoided while simultaneously promoting the internet as an important medium for increasing participation in sports.

The results show that the internet, as an important medium that affects residents' sport participation behavior, promotes residents' social communication, leisure and entertainment and learning. Currently, the rise of internet-based social media (WeChat, etc.), sports communities, live sports broadcasting platforms, and sports apps has created conditions for the construction of internet sports communities. Therefore, the establishment of internet sports communities should be encouraged to promote social communication, recreation and learning and facilitate the transmission and sharing of sports information to motivate residents to participate in sports.

As a kind of media, internet facilities need to provide internet sports services to promote residents' sport participation and improve the health levels of the population. Internet use affects participation in sports. Thus, age, marriage, and education differences should be actively targeted for internet popularization by increasing internet infrastructure investments and improving the internet infrastructure itself, such as through the implementation of China's “information into the village door project”, improving the capacity of internet services to less developed areas, and creating conditions for different groups using the internet. To improve internet infrastructure, differentiated and personalized internet sports services should be provided for young people, married groups and groups with low education levels to popularize fitness knowledge and scientifically guide vulnerable groups to participate in sports.

## Data availability statement

Publicly available datasets were analyzed in this study. This data can be found here: http://cgss.ruc.edu.cn.

## Author contributions

Z-hW conceived and designed the study. H-mZ performed the statistical analysis and wrote the paper. H-bX, E-kG, and JL revised the paper. All authors contributed to the article and approved the submitted version.

## Funding

This work was supported by the National Social Fund of China, Beijing, China (Grant No. 22ATY003) and the Decision Consulting Research Project of General Administration of Sport of China in 2019, Beijing, China (Grant No. 2019-C-09).

## Conflict of interest

The authors declare that the research was conducted in the absence of any commercial or financial relationships that could be construed as a potential conflict of interest.

## Publisher's note

All claims expressed in this article are solely those of the authors and do not necessarily represent those of their affiliated organizations, or those of the publisher, the editors and the reviewers. Any product that may be evaluated in this article, or claim that may be made by its manufacturer, is not guaranteed or endorsed by the publisher.
